# Solution structure and interaction with copper *in vitro* and in living cells of the first BIR domain of XIAP

**DOI:** 10.1038/s41598-017-16723-5

**Published:** 2017-11-30

**Authors:** Meng-Meng Hou, Panagis Polykretis, Enrico Luchinat, Xiao Wang, Shen-Na Chen, Hui-Hui Zuo, Yin Yang, Jia-Liang Chen, Yansheng Ye, Conggang Li, Lucia Banci, Xun-Cheng Su

**Affiliations:** 1 State Key Laboratory and Research Institute of Elemento-organic Chemistry, College of Chemistry, Collaborative Innovation Center of Chemical Science and Engineering (Tianjin), Nankai University, Tianjin, 300071 China; 2 Magnetic Resonance Center – CERM, University of Florence, 50019 Sesto Fiorentino, Florence Italy; 3 Department of Biomedical, Clinical and Experimental Sciences, University of Florence, 50134 Florence, Italy; 4 Key Laboratory of Magnetic Resonance in Biological Systems, State Key Laboratory of Magnetic Resonance and Atomic and Molecular Physics, National Center for Magnetic Resonance in Wuhan, Wuhan Institute of Physics and Mathematics, Chinese Academy of Sciences, Wuhan, 430071 China; 5 Department of Chemistry, University of Florence, 50019 Sesto Fiorentino, Florence Italy

## Abstract

The X-chromosome linked inhibitor of apoptosis (XIAP) is a multidomain metalloprotein involved in caspase inhibition and in copper homeostasis. It contains three zinc-binding baculoviral IAP repeats (BIR) domains, which are responsible for caspase interaction. Recently, it has been suggested that the BIR domains can bind copper, however high resolution data on such interaction is missing. Here we characterize by NMR the structural properties of BIR1 in solution, and the effects of its interaction with copper both *in vitro* and in physiological environments. BIR1 is dimeric in solution, consistent with the X-ray structure. Cysteine 12, located in the unfolded N-terminal region, has a remarkably low redox potential, and is prone to oxidation even in reducing physiological environments. Interaction of BIR1 with copper(II) results in the oxidation of cysteine 12, with the formation of either an intermolecular disulfide bond between two BIR1 molecules or a mixed disulfide bond with glutathione, whereas the zinc binding site is not affected by the interaction.

## Introduction

The X-chromosome linked inhibitor of apoptosis (XIAP) is a direct inhibitor of caspases and has been regarded as a potential target for therapy of cancer^1,2^. XIAP is a 497-residue cytoplasmic zinc-binding protein containing three baculoviral IAP repeats (BIR) domains at the N-terminal region, followed by an ubiquitin-associated domain (UBA) and a Really Interesting New Gene (RING) domain at the C-terminus. Each BIR domain contains a CCHC zinc binding motif, while the RING domain contains a CCCHCCCC motif that binds two zinc ions. XIAP was first recognized as an inhibitor of apoptosis due to its specific interactions with caspases 3 and 7, mediated by BIR2 domain, and caspase 9, mediated by BIR3^3–6^. BIR1 was found to be involved in the interaction with TAB1 in the NF-κB pathway^7^. Recently, additional roles of XIAP in receptor signaling, cell division, ubiquitin ligation and copper homeostasis have been reported^8^.

In human cells, the concentration of copper is tightly controlled by copper chaperones and imbalances of copper concentration results in diseases like Menkes or Wilson’s disease^9^. Recent studies have shown that XIAP is involved in cellular copper homeostasis and the direct interaction of XIAP with copper ions was reported^10–12^. It was also suggested that XIAP binds copper via the coordination of cysteines and it undergoes conformation changes upon copper binding^11^, resulting in decreased stability of XIAP. A later study showed that several cysteine residues in the BIR2 and BIR3 domains can coordinate copper(I), both at the zinc binding site and at additional surface sites^12^. Compared with BIR2 and BIR3, BIR1 was less studied, even though it has been reported by X-ray crystallography that BIR1 forms dimeric complex either in its free state or complexed with TAB1^7^. BIR1 forms a stable homodimer in the crystal structure, in which the BIR1 monomers are held together by electrostatic and hydrophobic interactions. However, the structural characterization of BIR1 in solution has not been reported and the function of the N-terminal residues (1–19), which were not observed in the crystal structure, has not been elucidated.

In the present study, the structural properties of BIR1 were characterized using high-resolution NMR spectroscopy *in vitro* and in different physiological environments, namely in the cytoplasm of *E. coli*, *X. laevis* oocytes and cultured human cells. The effect of copper addition in both oxidation and reduced states on BIR1 was then characterized. Wild-type BIR1 exists as a stable homodimer in solution, in line with the existing X-ray data. The dimerization can be impaired by two point mutations at the dimer interface, D71N/R72E, resulting in a well-folded monomeric protein. In-cell NMR experiments show that BIR1 interacts with cellular constituents in the cytoplasm of different cells, causing the loss of NMR signals from the well-structured residues. The N-terminal residues 1–19, which are absent in the crystal structure, are unstructured. No interaction between BIR1 and copper(I) was observed both *in vitro* and in-cell. Remarkably cysteine 12, which resides in a well conserved TCVP motif in the unstructured N-terminal tail among XIAP homologues, presents a very low redox potential (~−300 mV), and is found to react with copper(II) leading to the formation of either a disulfide-linked protein dimer or an adduct with glutathione *in vitro* and in-cell lysate.

## Results and Discussion

### Structural and dynamical characterization in solution of WT BIR1 and its D71N/R72E mutant

**Figure 1:**
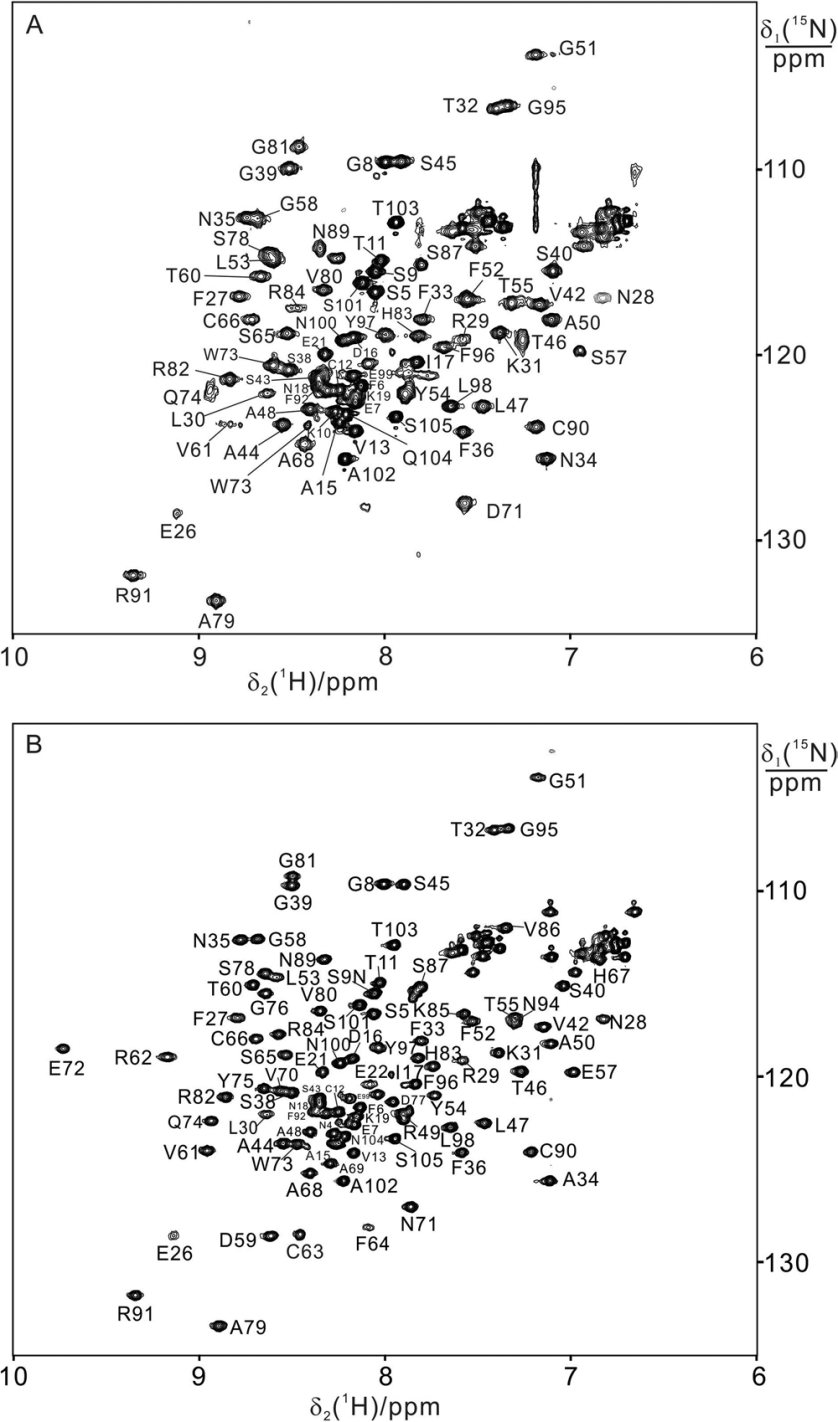
^15^N-HSQC spectra of BIR1 in solution. The NMR spectra were recorded for 0.3 mM WT BIR1 (1–105 aa) (**A**) and 0.3 mM D71N/R72E mutant of BIR1 (**B**) in 20 mM Bis-Tris buffer at pH 6.5 and 298 K with a proton frequency of 600 MHz. The cross-peaks with assignment were labeled.

The apo form of BIR1 (i.e. BIR1 lacking the zinc ion) is unfolded in solution as determined by ^15^N-HSQC spectra since all the cross-peaks show narrow dispersions and sharp linewidths, and hence in the present study all the protein samples were prepared by denature-refolding process in the presence of zinc ion (see experimental section). Wild-type BIR1 (WT BIR1) shows well dispersed resonances in the ^15^N-HSQC spectra, which are characteristic of a well-folded protein but characterized by quite large linewidths (Fig. 1A). ^15^N heteronuclear relaxation rates R_1_ and R_2_ of backbone amide nitrogen indicated different behaviors for various stretches of the protein, with the N- and C-termini characterized by high R_1_ and low R_2_, indicative of fast motions on the ns-ps time scale and the central part with the opposite pattern (Figure S1). Overall, the linewidths of most cross-peaks are larger than those expected from a monomeric polypeptide chain, suggesting that the protein exists as an oligomer or experiences conformation exchange in solution. The averaged R_2_/R_1_ ratios in the well-structured segments indicated a rotational correlation time (τ_c_) of 17.5 ns, consistent with a dimeric protein of 23 kDa. The large R_2_ values hampered the spectral assignment procedure based on standard triple resonance experiments. Therefore, we sought to design a mutant that would be monomeric in solution, in order to facilitate the NMR assignment. We then transferred the assignment of the mutant to the ^15^N-HSQC spectra of WT BIR1.

In the crystal structures of homodimeric BIR1^13^, D71 and R72 of one subunit form salt bridges with R82 and D77, respectively, of the other subunit. Additional hydrogen bonds are also present between T60-K85 and D71-K85. As the salt bridges involving D71 and R72 likely play an essential role in stabilizing the dimeric complex, the double-point mutant D71N/R72E was investigated. Compared to WT BIR1, D71N/R72E BIR1 gave rise to significantly narrower NMR signals (Fig. 1B), which permitted the assignment of most backbone resonances with the triple resonance NMR spectra HNCA, CBCA(CO)NH and NOESY-^15^N-HSQC. The cross-peaks in ^15^N-HSQC spectrum corresponding to residues of T2, F3, E20, F23, V24 and G56 were not assigned due to line broadening effects (Fig. 1). The ^15^N heteronuclear relaxation values R_1_ and R_2_ of backbone amide nitrogens have a similar pattern to those of WT BIR1 over the various sequence segments. The R_2_/R_1_ ratio gives a τ_c_ of 7.3 ns, consistent with the monomeric state.

NOESY-^15^N-HSQC spectra recorded for WT BIR1 and D71N/R72E indicated that both two protein constructs share similar secondary structural elements to the dimeric structure as determined by X-ray crystallography.^13^ The backbone assignment of D71N/R72E BIR1 was therefore transferred to WT BIR1 and cross-checked with 3D HNCA, CBCA(CO)NH and ^15^N-NOESY-HSQC spectra of WT BIR1. Residues E22, E25, A69, E72, Y75, G76, D77, K85, V86, F92, I93 and N94 could not be identified in the ^15^N-HSQC spectrum of WT BIR1, most likely due to line broadening effects.

**Figure 2:**
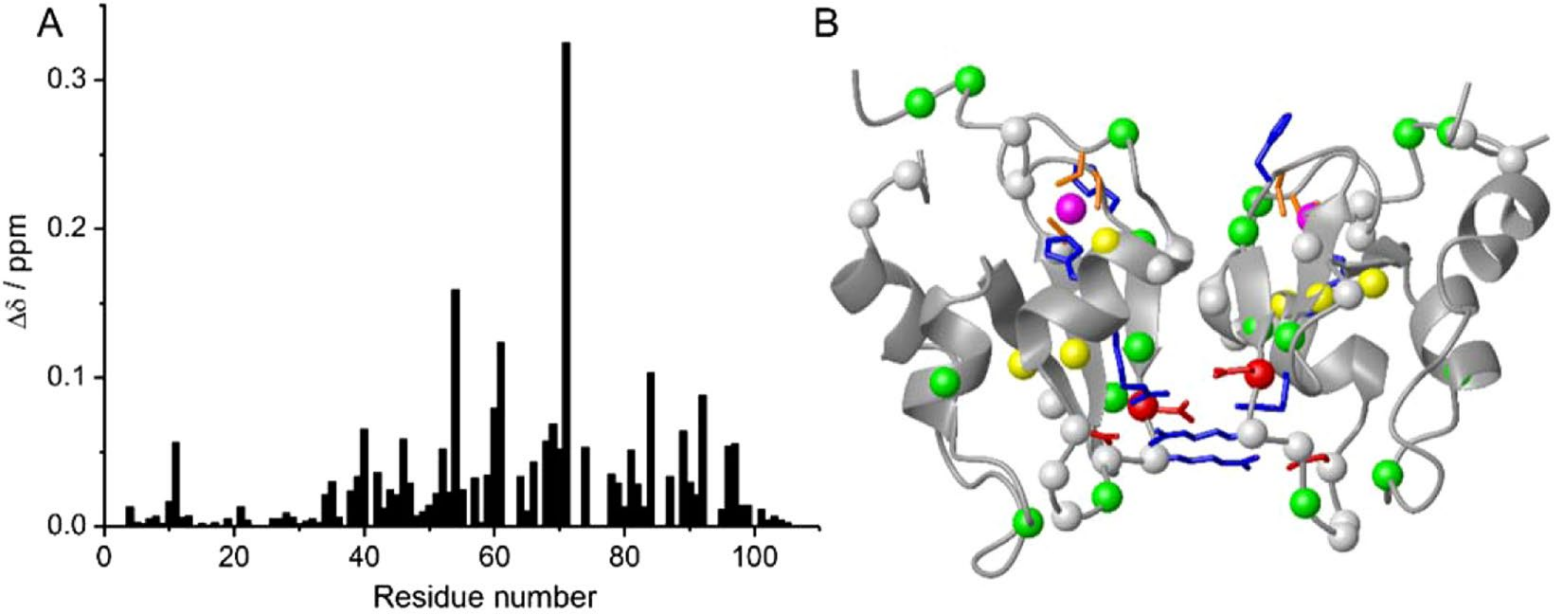
Chemical shift perturbations of D71N/R72E mutant on BIR1. (**A**) Plot of chemical shift perturbations caused by D71N/R72E mutation on BIR1 with the function of amino acid sequence. The chemical-shift differences between WT and D71N/R72E BIR1 were calculated as δ = ((Δδ_H_)^2^ + (Δδ_N_/10)^2^)^1/2^. (**B**) Chemical shift differences between WT BIR1 and D71N/R72E mutant plotted on the dimeric structure of BIR1 (PDB code: 2QRA)^13^, of which the Cα atoms are shown in red spheres with δ > 0.2 ppm, yellow spheres with 0.1 < δ ≤ 0.2 ppm, green spheres with 0.05 < δ ≤ 0.1 ppm, respectively. The unassigned Cα resonances in WT BIR1 were shown in grey spheres.

Chemical shift differences between the two forms were observed, as expected, for the residues vicinal to the homo-dimeric interface (Fig. 2A). Most unassigned residues in WT BIR1 are located at the dimer interface (Fig. 2B), suggesting that the dimeric BIR1 complex in solution experiences conformational exchange that broadens a number of residues in the ^15^N-HSQC spectra. The N-terminal residues 1–19, which are not observed in the X-ray structure^7,13^, generally produce narrower cross-peaks in the ^15^N-HSQC spectrum. Interestingly, the N-terminal residue T11 and C-terminal residues F96 and Y97 also show significant chemical shift difference between the dimeric and monomeric forms, suggesting that a dynamic contact between these residues may occur in the dimeric form in solution. The secondary structure prediction based on the backbone chemical shifts, obtained using TALOS+^14^, suggested that the stretches containing residues 3–16 and 97–105 are unstructured.

**Figure 3:**
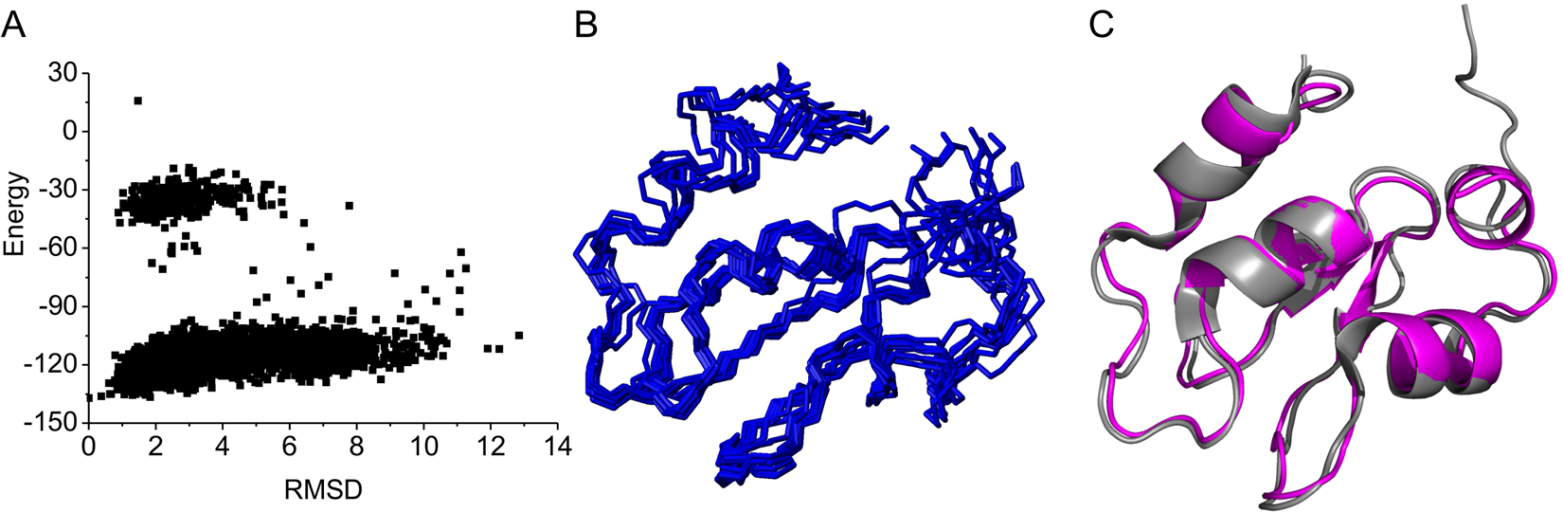
Solution structure of D71N/R72E BIR1 determined by CS-Rosetta. (**A**) Plot of Rosetta all atom energy versus ${{\rm{C}}}^{\forall }$ RMSD relative to the lowest energy model. (**B**) Ensemble structural representation of 10-lowest energy conformations of D71N/R72E BIR1 calculated by CS-Rosetta. (**C**) Structural comparison of the lowest energy solution structure of D71N/R72E BIR1 from CS-Rosetta calculation (magenta) and X-ray crystal structure of WT BIR1 (grey) (PDB code: 2QRA)^13^.

With the assignment of backbone resonances of the D71N/R72E mutant, we performed structural calculations using CS-Rosetta protocol^15^. The unstructured segments at the flexible N- and C-termini containing residues 1–22 and 97–105 were excluded from the calculations. The backbone chemical shifts of C^α^, C^β^, CO, HN and N were used in the CS-Rosetta calculations, and nicely converged structures for monomeric D71N/R72E mutant were determined (Fig. 3). The structures produced are well defined, with a mean pair-wise backbone RMSD among the 10 lowest-energy structures of 1.46 ± 0.71 Å. The lowest-energy structure has a 1.3 Å backbone root mean square deviation (RMSD) to the crystal structure of the BIR1 (PDB code: 2QRA)^13^. Consistent with the dimeric X-ray structure of WT BIR1^13^, the regularly structured elements produced well conserved short and long-range NOEs between HN-HN and HN-HA, indicative of α-helix and β-strands, in the NOESY-^15^N-HSQC spectrum recorded for D71N/R72E mutant, indicating the highly reliable CS-Rosetta structure.

### BIR1 is prone to oxidation in solution

Both WT BIR1 and the D71N/R72E mutant are prone to oxidation in solution as determined by NMR, mass spectrometry and gel filtration. When BIR1 is purified in absence of reducing agents, two species are observed in the ^15^N-HSQC spectra, where both WT and D71N/R72E BIR1 displayed additional cross-peaks. These additional cross-peaks disappeared upon addition of 2 equivalents of DTT, suggesting that oxidation of C12, the only solvent-exposed free cysteine in BIR1, may have occurred. The oxidized protein could be obtained by dialyzing the reduced protein sample in 20 mM Tris at pH 9 at 4 °C overnight. Comparison of the ^15^N-HSQC spectra reveals that the residues close to C11, including V13, experienced significant chemical shift changes upon oxidation (Figure S2). The oxidized species of D71N/R72E BIR1 was also characterized by MALDI-TOF mass spectrometry. Two main species were observed, corresponding to monomeric and dimeric D71N/R72E BIR1 (Figure S3). The latter species is absent in the sample treated with DTT. As the ^15^N NMR relaxation data show that D71N/R72E BIR1 is monomeric in solution, the dimeric species observed in the mass spectrum can be attributed to a covalent dimer formed by two D71N/R72E BIR1 monomers linked by an intermolecular disulfide bond.

Overall, our data indicate that the disulfide bond formed in the oxidized state involves C12, which resides in the flexible N-terminus. The ^15^N-HSQC spectrum of oxidized D71N/R72E BIR1 is essentially identical to that of the reduced form, suggesting that the structure of D71N/R72E BIR1 is not perturbed upon oxidation. Indeed, only the chemical shifts of T11 to V13 vary significantly, while the other residues are not essentially affected (Figure S4). In contrast, the ^15^N-HSQC spectrum of WT BIR1 is significantly affected, both in the dispersion and line-shape of the cross-peaks (Figures S2 and S4). In particular, residues 26–30 residing in the first α-helix are broader in the oxidized form, while the residues close to the dimer interface D59, R62 and C63 become narrower. Gel filtration chromatography showed that reduced and oxidized D71N/R72E mutant eluted at 31.5 and 28.8 min, respectively (Figure S5), consistent with the size increase between the monomeric protein and the covalent dimer. In contrast, the elution times of reduced and oxidized WT BIR1 were 30.2 and 31.5 min, respectively (Figure S5), suggesting that the formation of an intra-dimeric disulfide in the WT BIR1 dimer increases its compactness.

**Figure 4:**
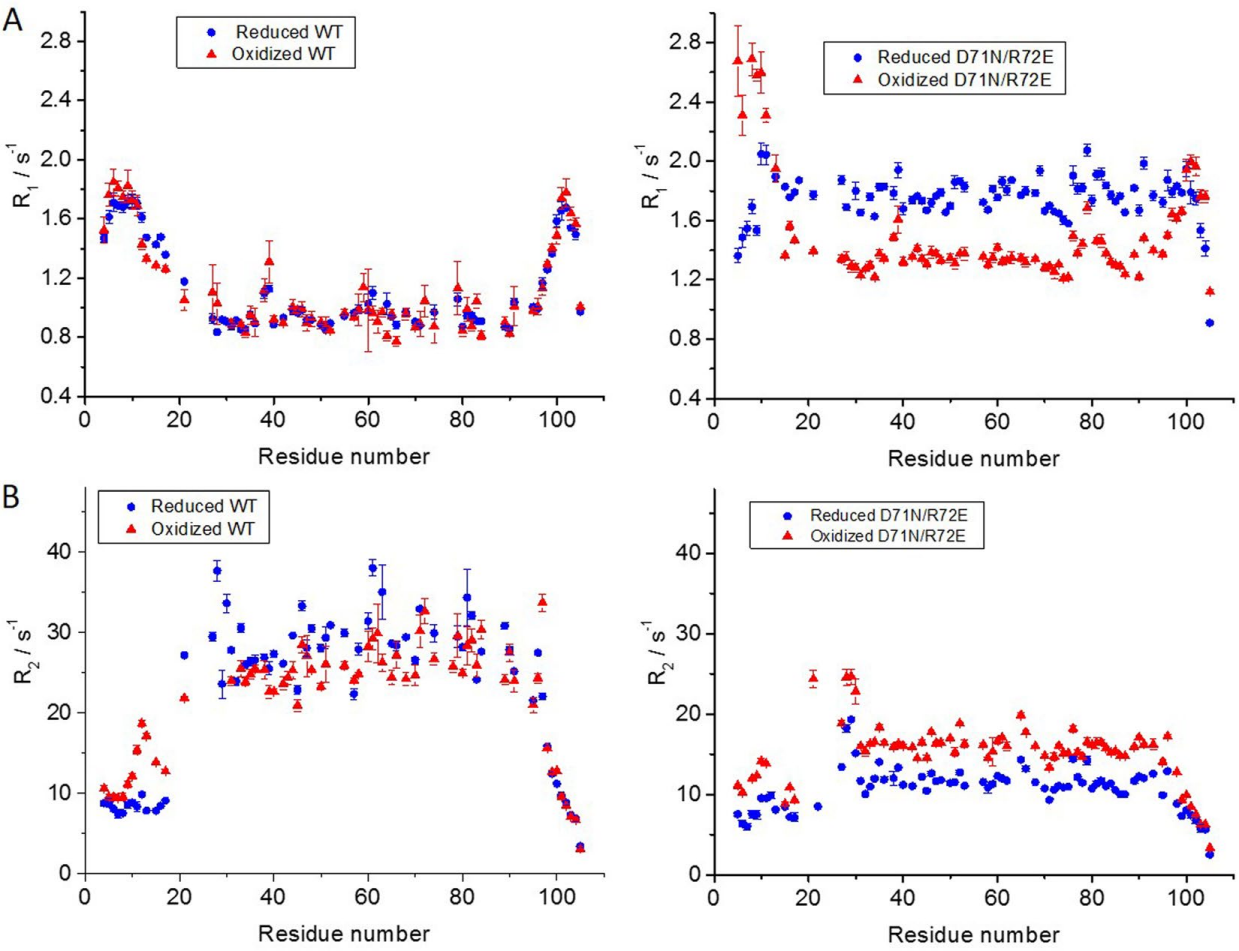
Relaxation rates of backbone ^15^N measured for BIR1 at 298 K in different conditions. (**A**) R_1_ of WT BIR1 and D71N/R72E mutant in the oxidized and reduced states. (**B**) R_2_ of WT BIR1 and D71N/R72E mutant. The protein concentration was about 0.3 mM in 20 mM Bis-Tris buffer at pH 6.5.

To further characterize the quaternary structure of oxidized BIR1, backbone ^15^N relaxation rates R_1_ and R_2_ were measured on oxidized WT BIR1 (Fig. 4). The latter presents a slightly smaller τ_c_ than the reduced state (16.4 ns vs 17.5 ns), confirming that the formation of an intramolecular disulfide bond between two C12 results in a more compact structure of the dimer. On the contrary, oxidation of the D71N/R72E mutant leads to an increase of τ_c_ from 7.3 ns to 11.8 ns. These relaxation results are in excellent agreement with the elution times from gel filtration experiments (Figure S5).

### Redox potential measurement

To determine the redox properties of BIR1, we measured the redox potential of C12 for both WT and mutant BIR1 by NMR, using DTT as the reducing reagent. The cross-peaks of V13 in the two forms were used to quantify reduced and oxidized BIR1. The determined standard redox potential of WT BIR1 and D71N/R72E mutant were −304 ± 7 and −296 ± 8 mV, respectively (Tables S1 and S2). Notably, this redox potential is quite low, and is close to that of the cytosol of human cells (−315 mV), as determined by the pool of GSH^16^.

To better simulate the intracellular conditions, we tested the reaction of GSH and oxidized BIR1 in solution, and found that GSH could not reduce oxidized BIR1 to its reduced form. In contrast, this reaction formed a mixed disulfide bond between GSH and BIR1, which was characterized by MALDI-TOF spectrometry (Figure S6). The complex of BIR1-GSH in ^15^N-HSQC spectra showed different spectral pattern from that of either oxidized or reduced of BIR1.

### BIR1 presents reduced form in living cells

**Figure 5:**
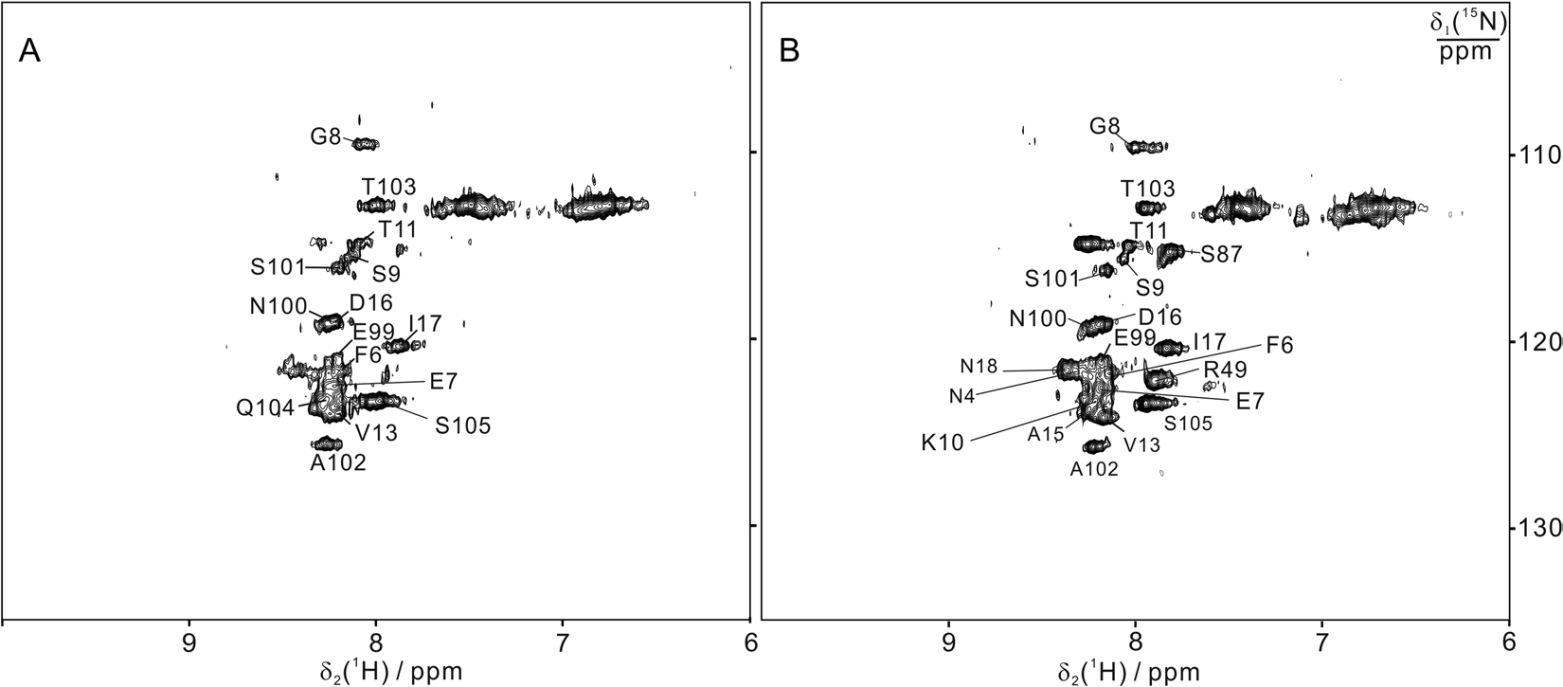
In cell ^15^N-HSQC spectra recorded in oocytes via microinjection at 298 K. (**A**) WT BIR1; (**B**) D71N/R72E mutant.

**Figure 6:**
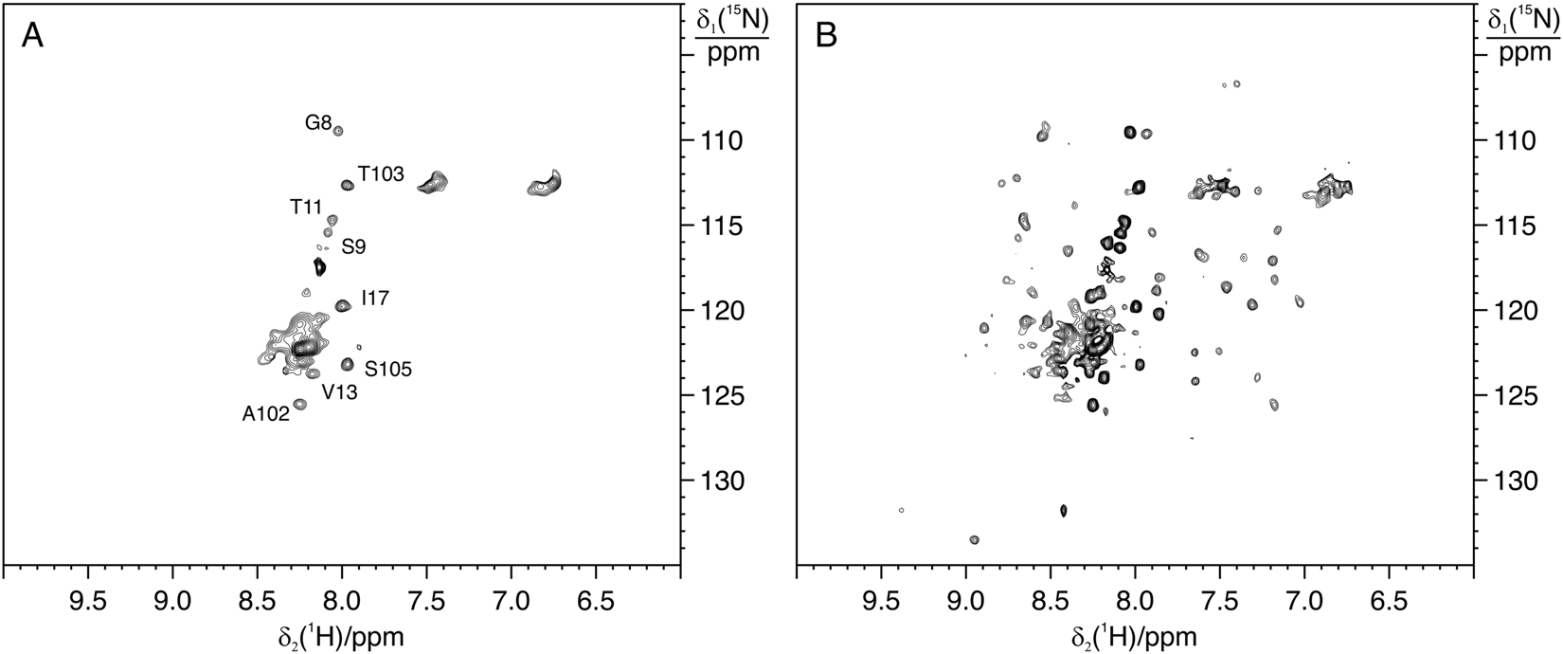
^15^N SOFAST-HMQC spectra of WT BIR1 in HEK293T cells and cell lysate. (**A**) On a sample of HEK293T cells expressing WT BIR1 and (**B**) on the corresponding cell lysate.

In order to assess whether the low redox potential of BIR1 affects its intracellular redox state, we carried out in-cell NMR experiments in living *E. coli* cells, *Xenopus laevis* oocytes, and cultured HEK293T cells, to determine the oxidation state of BIR1 in different cellular environments. In the various types of cells, the residues in well-structured regions of BIR1 did not give rise to detectable cross-peaks in the ^15^N-HSQC spectra, whereas a number of flexible residues in the N- and C-termini could be detected (Figs 5 and 6). In the cellular environment of *E. coli*, which has been reported to be more crowded than that of eukaryotic cells^17^, no signals from BIR1 are present, as all the observed cross-peaks arise from background intracellular species (Figure S7). These data indicate that, as is often the case for globular proteins^18,19^, BIR1 interacts with other cellular components, either with functional partners or non-specifically, causing broadening beyond detection of the NMR signals of the folded region.

**Figure 7:**
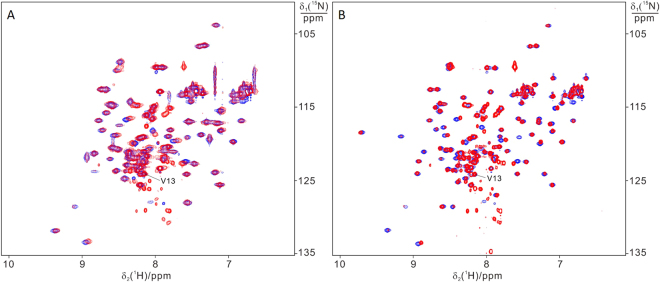
Comparison of ^15^N-HSQC spectra of BIR1 recorded for protein samples prepared from *in vitro* (blue) and in *E. coli* lysate (red). (**A**) WT BIR1; (**B**) D71N/R72E mutant. NMR spectra were recorded at 298 K with a proton frequency of 600 MHz.

To better assess the redox state of intracellular BIR1, we analyzed the NMR spectra of BIR1 in the cell lysates, which produced high quality NMR spectra for both WT BIR1 and D71N/R72E mutant, as the association of BIR1 with the cellular components was remarkably diminished upon cell lysis (Figs 6 and 7). Comparing ^15^N-HSQC spectra recorded *in vitro* with those of the cell lysates, all the residues showed negligible chemical shift changes with respect to the spectra of reduced BIR1, both in WT and D71N/R72E mutant. The oxidized form, which has clear chemical shift differences for the residues close to C12 (Figure S2), was not detected. These results suggest that in the cytoplasm, under basal conditions, the majority of BIR1 is in the reduced form. Nevertheless, the low redox potential of C12 likely makes the XIAP BIR1 domain prone to oxidation as a consequence of cellular stress phenomena.

### Effect of copper on BIR1 structure

**Figure 8:**
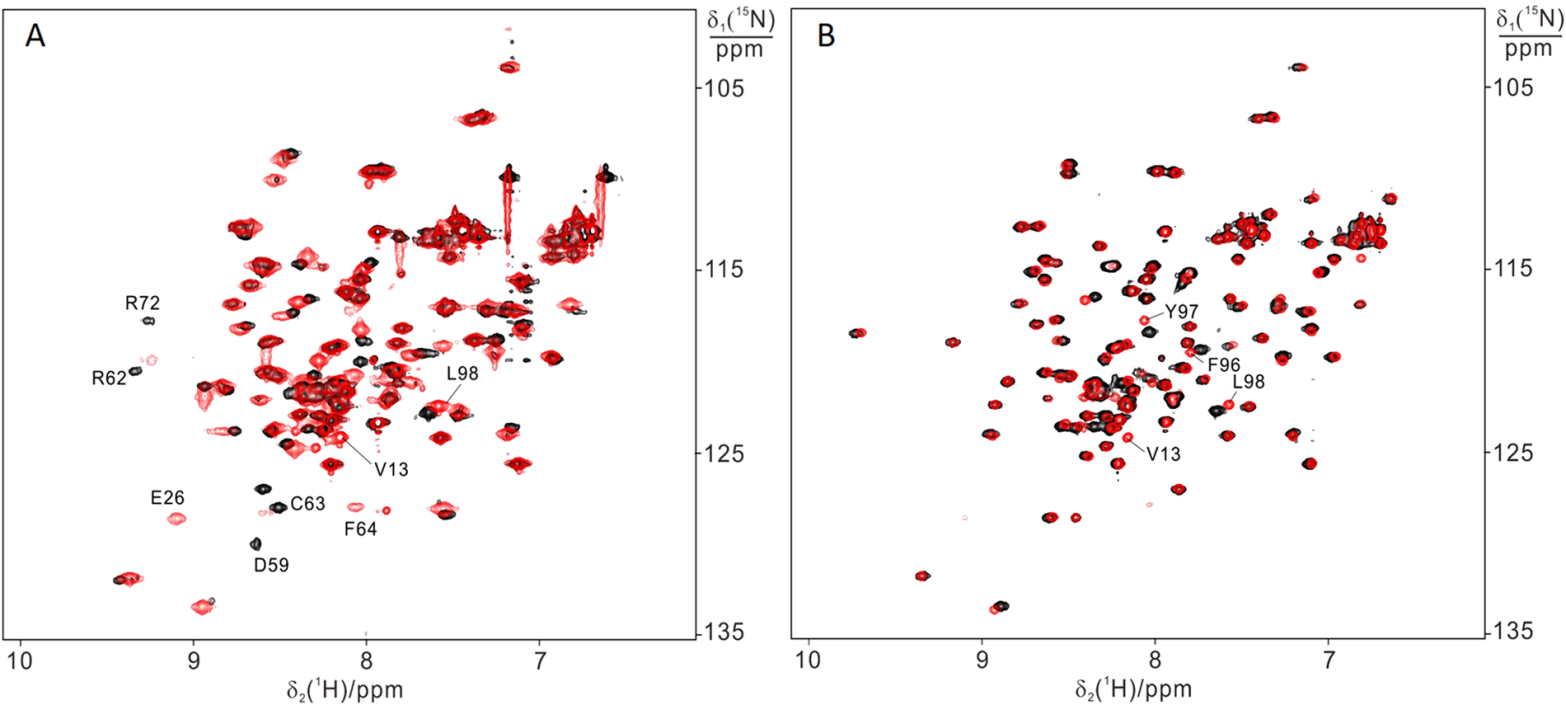
^15^N-HSQC spectra recorded for the solution of 0.2 mM protein in the absence (red) and presence (black) of 0.1 mM copper sulfate. (**A**) 0.2 mM WT BIR1; (**B**) 0.2 mM D71N/R72E. NMR spectra were recorded in 20 mM Bis-Tris, at pH 6.5 and at 298 K, with a proton frequency of 600 MHz.

XIAP has been shown to regulate cellular copper homeostasis, and recent data suggested a direct interaction between copper and XIAP^11,12^. It has been hypothesized that copper binding occurs either by displacement of Zn(II) in the zinc finger, or at additional sites on the surface. With these premises, we investigated the effect of copper on BIR1 *in vitro* at atomic resolution by NMR spectroscopy. The effect of Cu(II) was first characterized on both WT and mutant BIR1. As shown in Fig. 8, the addition of copper to the solution of either WT BIR1 and D71N/R72E mutant resulted in significant chemical shift perturbations.

New cross-peaks appeared, while some others were broadened in the ^15^N-HSQC spectra of both WT and D71N/R72E mutant (Fig. 8). As to WT BIR1, residues at the N-terminal segments containing T11, C12, V13 as well as E21, E22 and E26 experienced dramatic chemical shift changes upon addition of Cu(II) (Fig. 8A). In contrast to WT BIR1, D71N/R72E has less significant chemical shift perturbations as shown in Fig. 8B. It is noted that the residues in the zinc binding motif (C63, C66, H83 and C90) experienced no observable chemical shift changes or variations on cross-peak intensity upon titration with copper (Fig. 8B). Notably, the spectra of Cu(II)-treated BIR1 samples are almost identical to those of the oxidized state (shown in Figure S2). Chemical shift mapping analysis confirmed this observation (Figure S4). It is noted that some residues close to the zinc binding motif including R62, C63 and F64 also experienced minor chemical shift perturbations, which is probably an indirect consequence of the disulfide bond formation between C12 in the two subunits in WT BIR1. The oxidation of C12 was further confirmed by the fact that the addition of DTT to the mixture of Cu(II)-treated BIR1 samples reproduced the reduced state of BIR1 as determined by ^15^N-HSQC spectra (data not shown). In order to determine whether direct copper binding had occurred, the samples were washed to remove external copper ion with 20 mM Bis-Tris buffer and then analyzed by atomic absorption spectroscopy. No copper was determined in the protein samples, confirming that direct copper binding did not occur.

To further confirm that Cu(II) oxidizes BIR1 at C12, a triple mutant (C12A/D71N/R72E) was analyzed (Figure S8). Incubation of the triple mutant with Cu(II) did not produce any of the expected perturbations for the NMR signals in the ^15^N-HSQC spectrum (Figure S8), nor any difference in elution time as observed by gel filtration chromatography (Figure S8C). The interaction of BIR1 with Cu(II) resulting in oxidation of BIR1 rather than forming stable complex BIR1 is not buffer dependent, since both phosphate and Bis-Tris buffer produced almost identical chemical shift perturbations on protein signals as shown in the ^15^N-HSQC spectra (Figure S9).

Prompted by these interesting results, we analyzed the effect of Cu(I) on the structure of BIR1. The addition of one equivalent of Cu(I) to BIR1 in oxygen-free conditions caused the formation of precipitates, even at low (50 µM) protein concentration. NMR analysis of the residual soluble protein showed that most signals of both WT BIR1 and D71N/R72E mutant were broadened, except those of the residues at the flexible N- and C-termini (Figure S10). No chemical shift perturbation was observed, suggesting the absence of binding sites with high affinity for Cu(I).

Taken together, our data indicate that Cu(II) does not either substitute the zinc ion in the zinc finger motif or bind specifically with BIR1, but it serves as oxidant of C12 in formation of a disulfide bond. These results are in contrast with the previous findings stating that copper is capable of replacing zinc ion in the zinc finger motif or to bind to thiol groups of XIAP^11,12^.

The effect of Cu(II) on BIR1 was also investigated in the *E. coli* lysate and compared with the *in vitro* data. Addition of up to 0.5 mM Cu(II) into the cell lysate produced no detectable changes of NMR resonances as monitored by ^15^N-HSQC spectra. At higher copper concentrations, line broadening effects were observed for some cross-peaks, including C12. A few new cross-peaks were generated (Figure S11), which correspond to those arising from the BIR1-GSH complex, as the spectra are almost identical to those of BIR1 oxidized *in vitro* with excess of GSH. The formation of BIR1-GSH was confirmed by MALDI-TOF mass spectrometry (Figure S6), and is consistent with the high abundance of GSH in the cytoplasm and in the cell lysates.

The ability of Cu(II) to oxidize C12, both *in vitro* and in the cell lysates, forming either an inter-subunit disulfide bond or a complex with GSH suggests an interesting, previously unreported, property of XIAP. The sequence alignment of the first domain of XIAP from human, rat, cow, mouse, dog and *Xenopus laevis* (Figure S12) reveals that, in addition to the well-conserved residues forming the zinc finger motif, there is a conserved TCVP motif in four out of six species, suggesting that this motif may exert a kind of function that has not been explored yet.

## Summary

In this study, using high-resolution NMR spectroscopy, we show that the BIR1 domain of human XIAP forms a homodimer in solution, in accordance with the X-ray crystal structure. The dimer is destabilized by removing two salt bridges at the binding interface, resulting in the monomeric D71N/R72E mutant. In both WT and mutant BIR1, the first 19 residues at the N-terminus are flexible in solution. These flexible residues are invisible in the X-ray structures both in its free state and in complex with TAB1. A TCVP motif was identified in the N-terminal unstructured segment, which is conserved among XIAP orthologues and likely plays an important role in the interaction with copper. Cysteine 12 in the TCVP motif is prone to oxidation, resulting in the formation of a disulfide bond between two BIR1 subunits, or in the formation of a mixed BIR1-GSH disulfide bond in a physiological environment. The redox potential of C12, ~−300 mV, is lower than the generally known cysteine-related reductases, and comparable to that of the human cytosol. Considering the suggested role of XIAP in copper homeostasis, we further investigated the interaction of BIR1 with copper. In contrast to previous findings, the zinc ion bound to BIR1 is not replaced by Cu(II) or Cu(I), and the zinc finger domain has negligible changes upon interaction with copper. Cu(I) binding occurs non-specifically and with low affinity, therefore it is unlikely that BIR1 directly binds copper in a physiological environment. Instead, Cu(II) is able to oxidize C12 both in buffer and in the cell lysate.

These findings suggest a novel regulation mechanism of XIAP that deserves to be further investigated, in which C12 oxidation would modulate the biological roles of XIAP in response to intracellular stimuli or changes in the redox environment.

## Materials and Methods

### Protein expression and purification

The cDNA encoding the sequence (1–105) of the XIAP, following the previous report^7^, was amplified and cloned into the pET-3a expression vector. The D71N/R72E and D71N/R72E/C12A mutants were made by PCR-based quick-change mutagenesis method and all the plasmids were confirmed via DNA sequencing. The recombinant vector was transformed into *Escherichia coli* BL21(DE3) CodonPlus strain, and single colonies were selected for ampicillin and chloramphenicol resistance.

^15^N-labeled protein was prepared by growing cells in M9 medium following an optimized high cell-density protocol^20^. The bacteria were first grown in LB medium and when the cell density reached 0.6 O.D. at 600 nm, the cells were gently collected and washed with MilliQ water. The cells were then transferred in the M9 medium containing 160 mM Na_2_HPO_4_, 40 mM KH_2_PO_4_, 2 mM MgSO_4_, 0.1 mM CaCl_2_, a trace metal mixture^21^, 1% ^12^C-glucose (or ^13^C-glucose for ^13^C for labeling), ^15^NH_4_Cl (1 g/mL) and 0.1 mM ZnCl_2_. The cells were allowed to recover by incubation at 30 °C for 1 h. Protein expression was subsequently induced by addition of isopropyl β-D-1-thio-galactopyranoside (IPTG) to a concentration of 1 mM. After 10 h incubation, the cells were harvested. Large fractions of BIR1 protein were shown as being precipitated in the inclusion bodies during the over-expression. The cell lysate was centrifuged and the precipitates were solubilized in a solution of 8 M urea, 20 mM dithiothreitol (DTT) and 20 mM PBS (pH 8). The protein was refolded by using a multistep dialysis against solutions containing 6 M urea, 20 mM PBS, pH 8.0, 0.1 mM ZnCl_2_, and 0.2 mM DTT; 4 M urea, 20 mM PBS, pH 8.0, 0.1 mM ZnCl_2_ and 0.2 mM DTT; 2 M urea, 20 mM PBS, pH 8.0, 0.1 mM ZnCl_2_ and 0.2 mM DTT; and finally 20 mM PBS, pH 8.0, 0.1 mM ZnCl_2_ and 0.2 mM DTT. The protein was purified through DEAE column followed by Superdex-75 gel filtration. The buffer was then exchanged with 20 mM Bis-Tris at pH 6.5.

### NMR experiments and protein assignment

All NMR spectra were recorded at 298 K on a Bruker AV600 NMR spectrometer equipped with a QCI-cryoprobe unless noted elsewhere. The triple-resonance NMR experiments were performed on protein samples at concentration of 1 mM ^15^N,^13^C-BIR1 (both for WT BIR1 and D71N/R72E mutant) in 20 mM Bis-Tris buffer at pH 6.5. All the NMR spectra were processed with the Bruker Topspin 2.1 and analyzed using NEASY program in CARA package^22^ and Sparky^23^.

The backbone resonance assignments were obtained from the analysis triple resonances of 3D HNCA, HNCACB and CBCA(CO)NH together with NOESY-^15^N-HSQC spectra and crystal structure of BIR1^7^. Wild type BIR1 has broad NMR signals and the backbone assignment was achieved with the 3D crystal structure of BIR1^7^ and the completed assignments of BIR1 D71N/R72E mutant, since both wild type BIR1 and D71N/R72E mutant present similar long- and intermediate-range NOEs according to the crystal structure.

Backbone ^15^N-relaxation data of wild type BIR1 and D71N/R72E mutant were recorded at 298 K using standard experiments^24^. The relaxation delays were 17, 34, 51, 68, 85, 102, 119 ms in the *R*_2_ experiment and 3, 30, 150, 400, 550, 700, and 900 ms in the *R*_1_ experiment. The complete set of *R*_1_ and *R*_2_ relaxation data was recorded in about 18 h per protein sample, using *t*_1max_ = 45 ms and *t*_2max_ = 110 ms for each spectrum.

### Structure Calculations by CS-Rosetta

Software CS–Rosetta 3.2 protocol^15^ was applied to determine the 3D structure of D71N/R72E mutant using the backbone chemical shifts of ^15^N, ^1^HN, ^13^Cα, ^13^C_β_, and ^13^CO (318 in total). Homology CS-RosettaCM calculations were performed based on the crystal structure of BIR1^7^; Zinc(II) was not added during the structural calculation. The convergence of the predicted models was established on the basis of plots of energy vs. backbone RMSD to the lowest energy (‘best’) structure following the established protocol^15^.

### Oxidation of BIR1

The solution of 0.3 mM BIR1 (wild type or D71N/R72E) was dialyzed in 20 mM Tris buffer at room temperature, at pH 8.0, for 12 hours and the oxidation process was monitored by ^15^N-HSQC spectra. Analytical gel filtration measurements were performed on a Superdex 75 10/200 GL column (GE Healthcare Life Sciences) attached to an UPC-900 AKTA FPLC system in 20 mM Bis-Tris pH 6.5. Atomic absorption was analyzed on the Hitachi 180-80 Polarized Zeeman Atomic Absorption Spectrometer.

### Redox potential measurement

The redox potential of BIR1 was measured by NMR-based titration of the protein with DTT in 100 mM PBS buffer at pH 7.0. ^15^N-labeled oxidized BIR1 samples (0.32 mM for BIR1 D71N/R72E and 0.28 mM for WT BIR1) were incubated with increasing concentrations of reduced DTT. At each titration point, one ^15^N-HSQC spectrum of BIR1 was recorded to measure peak heights for the oxidized and reduced populations of the BIR1. To make sure that the reactions have reached equilibrium, the mixtures in NMR tubes were incubated at pH 7.0, at room temperature for 2 h prior to data acquisition. To reduce air oxidation, all solutions were degassed and the NMR tubes were purged with nitrogen. The BIR1 redox reactions for WT BIR1 and D71N/R72E can be described as the following:1
2when BIR1_ox_ was incubated with varying concentration of reduced DTT, the fractions of reduced BIR1_red_, oxidized BIR1_ox_, reduced DTT_red_ and oxidized DTT_ox_ in equations (1) and (2) were determined as following. The concentration of reduced and oxidized BIR1 under equilibrium condition in equations (1) and (2) was determined by measuring the cross-peak intensity of residues in the ^15^N-HSQC spectra following a linear relationship of cross-peak intensity versus the concentrations of protein, respectively. For WT BIR1, in equation (1), the concentration of oxidized DTT, DTTox, equals the concentration of reduced BIR1, BIR1red, under equilibrium condition. As to D71N/R72E mutant, the concentration of DTTox, is half of that of BIR1red, under equilibrium condition in equation (2). In the following expressions, [DTTred, total] represents the initial concentration of reduced DTT, and [BIR1ox, total] the initial concentration of oxidized BIR1, respectively.The standard potential E in equation (1) can be written as Nernst equation:

$${E}^{o}={E}_{DTT}^{0}-{E}_{BIR1}^{0}=-\frac{RT}{nF}\text{lnQ},$$

where Q is the equilibrium constant in equations (1) and (2), R the gas constant (8.315 J·K^−1^·mol^−1^), T the absolute temperature (298.15 K), n the number of transferred electrons (2) and F the Faraday constant (9.649 × 10^4^ C·mol^−1^). Q can be rewritten as:

$${\rm{Q}}=\frac{[BIR{1}_{red}][DT{T}_{ox}]}{[BIR{1}_{ox}][DT{T}_{red}]},\,{\rm{for}}\,{\rm{equation}}\,(1)\,{\rm{and}}$$

$${\rm{Q}}=\frac{{[BIR{1}_{red}]}^{2}[DT{T}_{ox}]}{[BIR{1}_{ox}][DT{T}_{red}]}\,{\rm{for}}\,{\rm{equation}}\,(2),$$

respectively, under equilibrium condition. The concentration of reduced and oxidized BIR1, and reduced and oxidized DTT can be determined following the linear relationship of cross-peak intensity of NMR signals to the concentration of protein, which is determined independently. Knowing the standard potential of DTT, E^0^_DTT_ −323 mV^25^, the standard potential of BIR1 can therefore be determined.

### In-cell *E. coli* NMR experiment

The expression system was the same as described above. The cells containing BIR1 vector were first grown in 200 mL LB until absorbance at 600 nm (OD A600) reached about 0.8 and the cells were gently collected by centrifuge (3000 rpm for 10 minutes). The cells were washed with MilliQ water and then resuspended in 50 mL M9 medium with ^15^N-NH_4_Cl as sole nitrogen source. After 20 minutes, the culture was induced with isopropyl β-D-thiogalactopyranoside (IPTG) to a final concentration of 1 mM. After 3 h, a 10 mL aliquot was pelleted at 3000 rpm for 10 min at 4 °C. The pellet was then resuspended in 0.5 mL of 20 mM Bis-Tris, pH 6.4 and 10% D_2_O. The remaining 40 mL culture was incubated with shaking for 5 h before processing as described above.

After obtaining in-cell NMR spectra, the cells were pelleted immediately by centrifuge at 5000 rpm for 5 min at 4 °C. The supernatant was checked for protein leakage by NMR. The resulting cell pellets were resuspended in 1 mL 20 mM Bis-Tris buffer at pH 6.5 and lysed with sonication. The cell lysate was harvested by centrifugation at 14000 g for 10 min at 4 °C and the supernatant was applied for NMR measurement.

### Preparation of bacterial cell lysate

*E. coli* cells were first grew in 600 mL LB media and then in 100 mL M9 medium. The cells were collected and washed with lysate buffer (20 mM Bis-Tris, pH 6.5 free from reducing reagent like DTT or TCEP). Then the cells were mixed with 2 mL lysate buffer and the mixture was sonicated in an ice bath with cooling intervals between sonication bursts. The sonication cycle was repeated until the suspension became partially clear. Then the solution was centrifuged at 12000 rpm for 15 min at 4 °C. The supernatant was carefully transferred to an NMR tube for measurement.

### In *Xenopus laevis* oocytes NMR measurement of BIR1

The in-cell NMR sample preparation was based on the previously established protocol^26–28^. The solution of 0.2 mM ^15^N-labeled BIR1 samples (about 550 µL) was lyophilized and then dissolved in 30 µL MilliQ water without further adjusting the pH. The protein concentration for micro-injection was typically about 3.6 mM. Each oocyte was injected with ~30 nL of ~3.6 mM ^15^N-enriched proteins via an IM-300 microinjector (Narishige Co. Ltd., Tokyo, Japan). Injected oocytes (about 200) were put into a 5 mm Shigemi NMR tube containing ND96 buffer (96 mM NaCl, 2 mM KCl, 1.8 mM CaCl_2_, 1 mM MgCl_2_, 5 mM HEPES, pH 7.4) plus 10% D_2_O. After the in-cell NMR experiments, about 200 μL of buffer above the oocytes was suctioned for a protein leakage test. No leakage was observed after NMR measurement.

### In-cell NMR in HEK293T cells

The gene of WT BIR1 (encoding the a.a. 1–105) was cloned into the pHLsec vector^29^ between EcoRI and XhoI restriction sites, and was verified by DNA sequencing. Samples for in-cell NMR were prepared following a reported protocol^30–32^. Briefly, adherent HEK293T cells were grown on uncoated 75 cm^2^ plastic flasks at 37 °C in 5% CO_2_ atmosphere, and were maintained in Dulbecco’s Modified Eagle’s medium (DMEM; high glucose, D6546, Sigma) supplemented with L-glutamine, antibiotics (penicillin and streptomycin) and 10% FBS (Gibco). Cells were transiently transfected with the pHLsec plasmid containing BIR1, using polyethylenimine (PEI) in the ratio 1:1, in ^15^N labelled media (BioExpress6000), supplemented 2% FBS and Zn(II) as ZnSO_4_, to a final concentration of 10 μM. After 48 h, the cells were collected and placed in a 3 mm Shigemi NMR tube. After the NMR experiments, the cell lysates were obtained by freeze-thaw lysis in PBS buffer, followed by centrifugation at 15000 g. NMR experiments on intact cells and lysates were recorded at 298 K at a 950 MHz Bruker Avance spectrometer equipped with a TCI CryoProbe.

### Interaction of BIR1 with copper

The interaction of BIR1 with copper(II) was analyzed by recording the ^15^N-HSQC spectra. ^15^N-HSQC spectra was recorded with addition of copper sulfate from a 10 mM stock solution into the solution of 0.2 mM BIR1 (either wild type or D71N/R72E). A number of ^15^N-HSQC spectra were recorded at the molar ratio of [Cu^2+^]/[protein] 0, 0.2, 0.6, 0.8, 1.0 and 1.2, respectively, in 20 mM Bis-Tris at pH 6.5. For comparison, mixture of ascorbic acid and copper sulfate (molar ratio 10:1) was added into the same concentration of BIR1, and ^15^N-HSQC spectra were recorded accordingly. The precipitates formed during the addition of copper were characterized by Atomic Absorption Spectroscopy (AAS).

To investigate the interaction of BIR1 with copper(I), BIR1 proteins were treated with tetrakis(acetonitrile)copper(I) hexafluorophophate, [Cu(I)(CH_3_CN)_4_]PF_6_, which was made of 50 mM stock solution in acetonitrile. The samples of 0.15 mM ^15^N-labeled BIR1 proteins in the absence and presence of one equivalent of Cu(I) were made in a glove box without oxygen, and were analyzed by ^15^N-HSQC spectra.

### Interaction of BIR1 with copper(II) in cell lysates

To investigate the interaction of BIR1 and copper (II) in a cell lysate, *E. coli* cells expressing ^15^N labeled BIR1 (wild type or D71N/R72E) were lysed in 20 mM Bis-Tris buffer at pH 6.5 without DTT. The obtained lysate was centrifuged and the supernatant was analyzed by NMR. A solution of copper sulfate was then added to the lysate and ^15^N-HSQC spectra were recorded subsequently.

## Electronic supplementary material

Supplementary Information
