# Supplementary material for: Solution structure and interaction with copper in vitro and in living cells of the first BIR domain of XIAP
Source: Sci Rep. 2017 Nov 30;7:16630. doi: 10.1038/s41598-017-16723-5 (PMC5709467; doi:10.1038/s41598-017-16723-5)
Supplement: Supplementary file 1 — Supplementary Information [file 41598_2017_16723_MOESM1_ESM.pdf]

Solution structure and interaction with copper *in vitro* and in living cells of the first BIR domain of XIAP

Meng-Meng Hou[a,*], Panagis Polykretis[b,*], Enrico Luchinat[b,c,*], Xiao Wang[a*], Shen-Na Chen[a*], Hui-Hui Zuo[a*], Yin Yang[a], Jia-Liang Chen[a], Yansheng Ye[d], Conggang Li[d], Lucia Banci[e], Xun-Cheng Su[a]

[a]State Key Laboratory and Research Institute of Elemento-organic Chemistry, College of Chemistry, Collaborative Innovation Center of Chemical Science and Engineering (Tianjin), Nankai University, Tianjin 300071, China.
[b]Magnetic Resonance Center – CERM, University of Florence, 50019 Sesto Fiorentino, Florence, Italy.
[c]Department of Biomedical, Clinical and Experimental Sciences, University of Florence, 50134 Florence, Italy.
[d]Key Laboratory of Magnetic Resonance in Biological Systems, State Key Laboratory of Magnetic Resonance and Atomic and Molecular Physics, National Center for Magnetic Resonance in Wuhan, Wuhan Institute of Physics and Mathematics, Chinese Academy of Sciences, Wuhan 430071, China.
[e]Department of Chemistry, University of Florence, 50019 Sesto Fiorentino, Florence, Italy.
* These authors have contributed equally to this work.

Correspondance to: Lucia Banci, banci@cerm.unifi.it and Xun-Cheng Su, xunchengsu@nankai.edu.cn

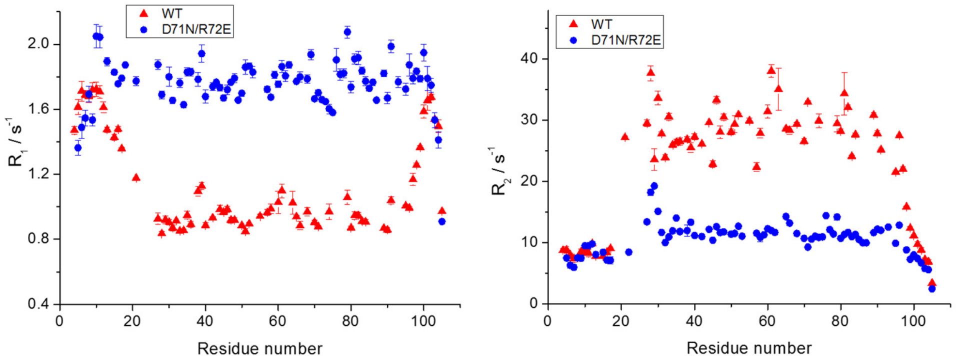

Figure S1. Relaxation measurements of BIR1. ^{15}N-relaxation rates measured for wild type BIR1 and D71N/R72E mutant at 298 K in 20 mM Bis-Tris buffer at pH 6.5, with a proton frequency of 600 MHz.

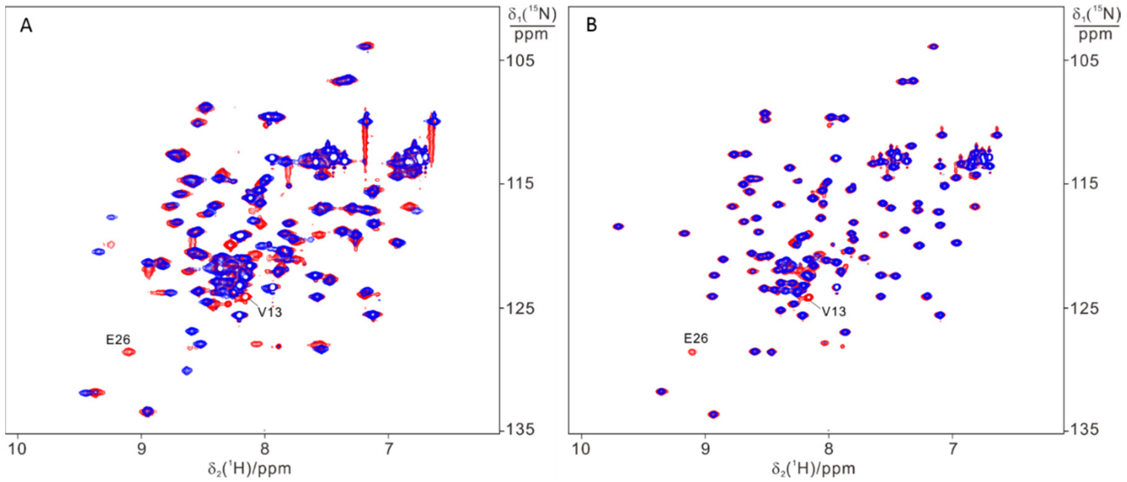

Figure S2. Comparison of oxidized and reduced BIR1. Superimposition of ^{15}N-HSQC spectra recorded for 0.2 mM ^{15}N-BIR1 in the reduced (red) and oxidized (blue) form. (A) WT BIR1; (B) D71N/R72E mutant. NMR spectra were recorded in 20 mM Bis-Tris at pH 6.5 and 298 K, with a proton frequency of 600 MHz. It is noted that in the oxidized state, the residues close to C11 including V13 experienced significant chemical shift changes and the chemical shift perturbations with the function of amino acid sequence are shown in Figure S4.

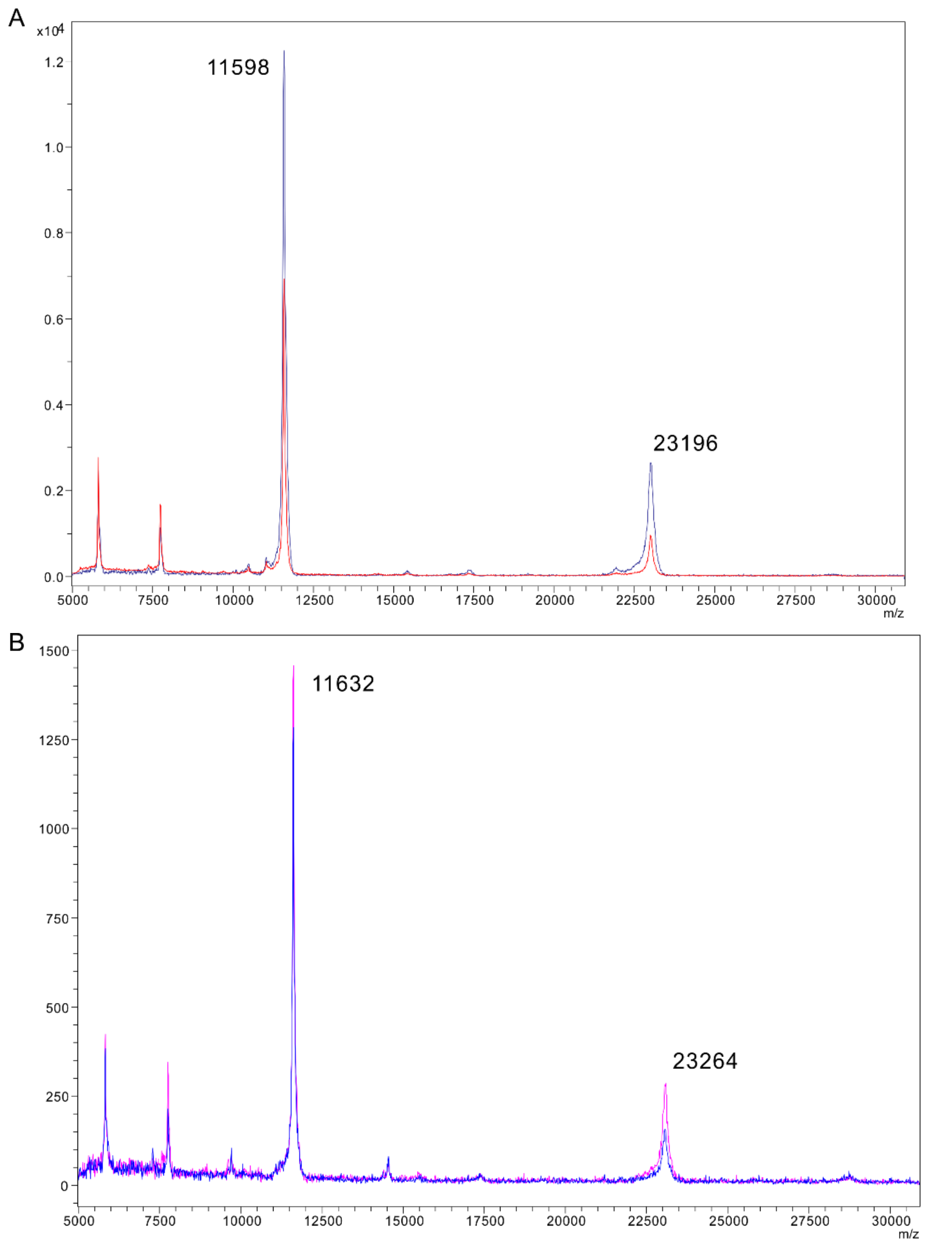

Figure S3. MALDI-TOF mass spectra recorded for the mixture of reduced and oxidized BIR1 and oxidized BIR1. (A) D71N/R72E: mixture of reduced and oxidized states (red) and oxidized state (blue). (B) WT BIR1: mixture of reduced and oxidized states (blue) and oxidized state (magenta). The spectra were recorded using matrix of α-cyno-4-hydroxycinnamic acid in water:CH_3CN (1:1) and 00.1% CF_3COOH, in which condition the zinc ion was released from protein due to low pH (about 3.5) and only free polypeptide was determined.

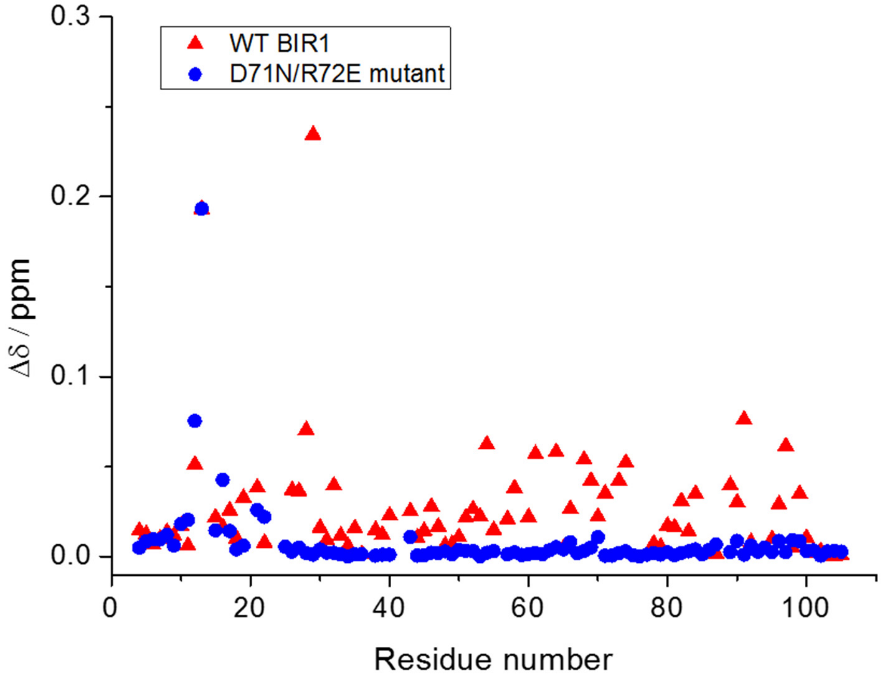

Figure S4. Chemical shift changes of BIR1 between the oxidized and reduced states with the function of amino acids. Chemical shift differences were calculated as $\Delta\delta = \mathrm{Sqrt}[(\Delta\delta_H)^2 + (\Delta\delta_N/10)^2]$, where $\Delta\delta_H$ and $\Delta\delta_N$ are the backbone amide chemical shift differences in the hydrogen and nitrogen dimension, respectively.

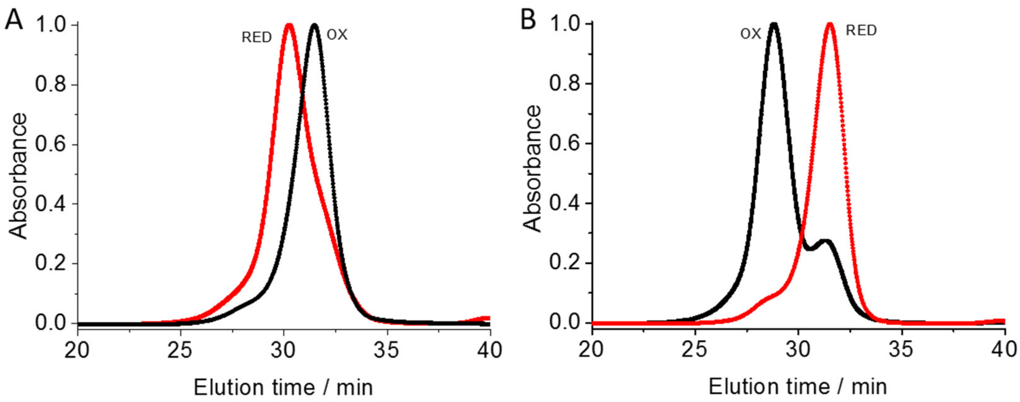

Figure S5. Gel filtration analysis of reduced and oxidized BIR1. Gel filtration profiles of reduced (red) and oxidized (black) WT BIR1 (A) and D71N/R72E (B), of which the absorption was monitored by UV lamp at 280 nm.

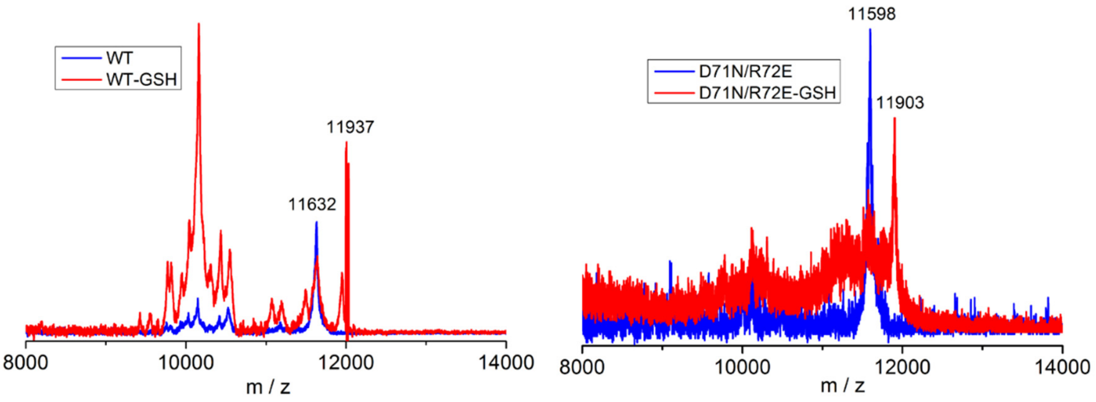

Figure S6. MALDI-TOF mass spectra analysis of oxidized BIR1 after treatment with GSH. Spectra were recorded for the reduced 0.1 mM WT BIR1 (left) and D71N/R72E mutant (right) and mixture of 0.1 mM oxidized WT BIR1 and D71N/R72E mutant and 0.2 mM GSH. The calculated molecular mass difference of 305 mass units between BIR1 and BIR1-GSH is identical to the difference of masses observed.

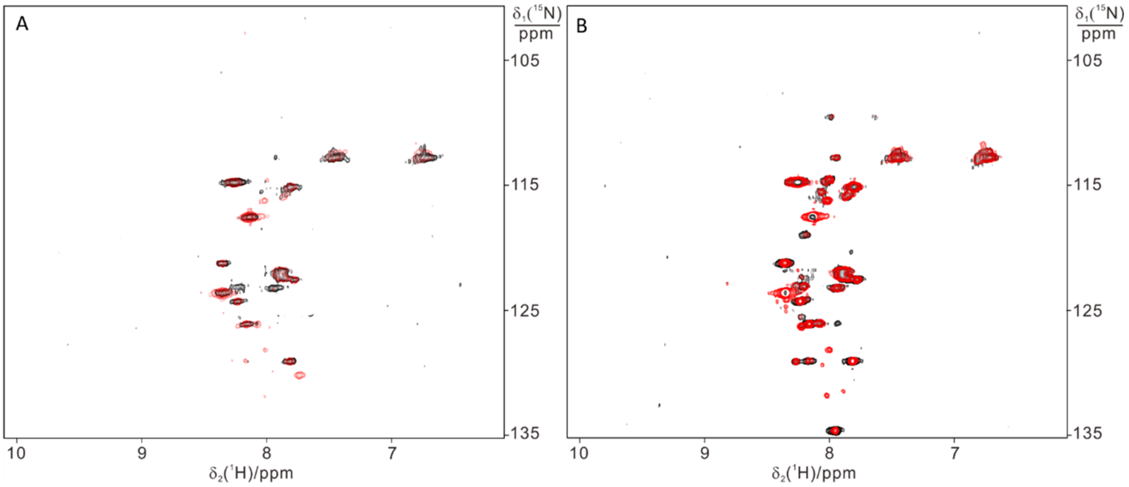

Figure S7. ^{15}N-HSQC spectra recorded in-cell living *E. coli* cells. The cells were collected before (red) and after (black) IPTG induction for NMR measurements. A) WT BIR1; B) D71N/R72E mutant. The NMR spectra were recorded at 298 K and in 20 mM Bis-Tris buffer at pH 6.5. In *E. coli* cells, even the NMR signals of the flexible regions of BIR1 were not determined.

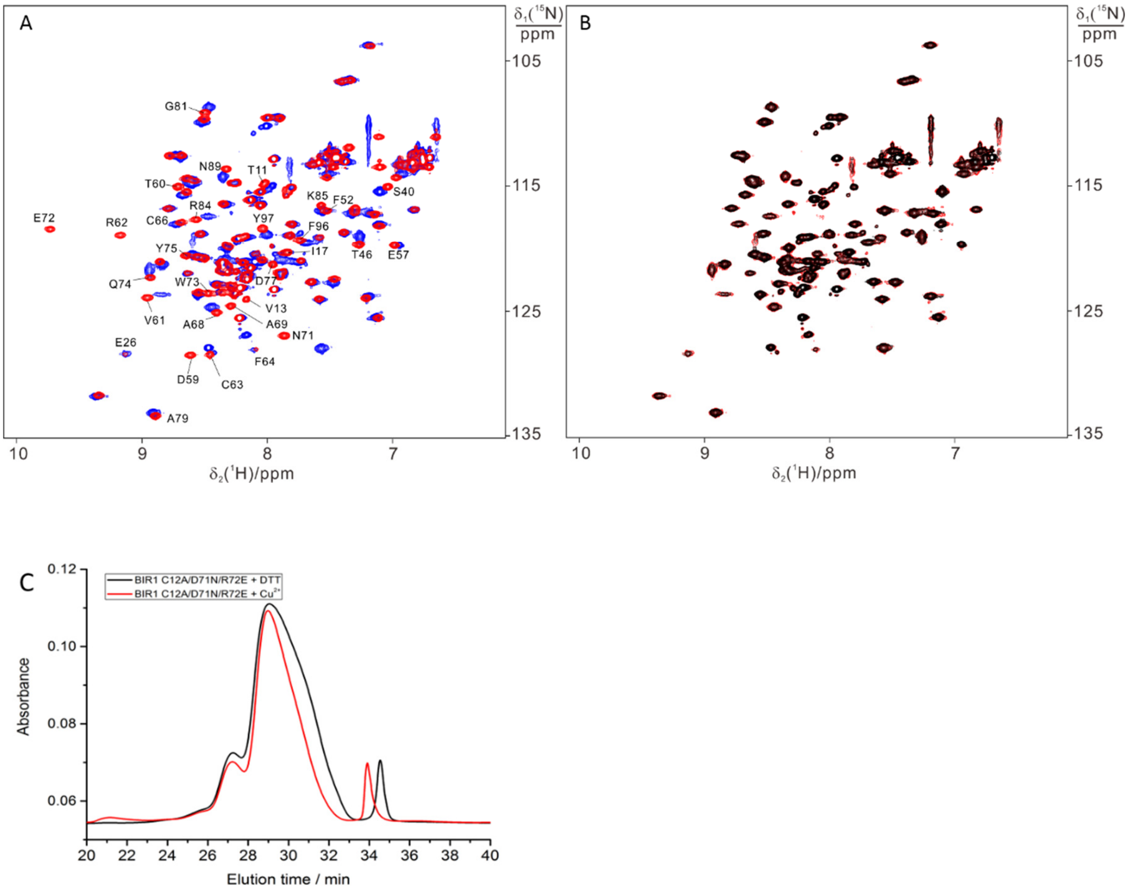

Figure S8. Comparison of BIR1 mutants in interaction with copper sulfate. (A) 0.2 mM D71N/R72E mutant (red) and 0.2 mM C12A/D71N/R72E mutant (blue), of which residues with large chemical shifts changes are labeled for D71N/R72E mutant. (B) 0.2 m M C12A/D71N/R72E mutant in the absence (red) and presence (black) of 0.5 mM copper sulfate. NMR spectra were recorded in 20 mM Bis-Tris at pH 6.5 and 298 K with a proton frequency of 600 MHz. (C) Gel filtration analysis of C12A/D71N/R72E mutant in interaction with copper (II). Gel filtration profiles of C12A/D71N/R72E mutant in the presence of 5 equivalents of DTT (black) and 0.6 equivalent of copper sulfate (red), respectively, of which the absorption was monitored by UV lamp at 280 nm. The protein samples treated with DTT or copper sulfate were incubated for 5 hours at room temperature prior to gel filtration experiment.

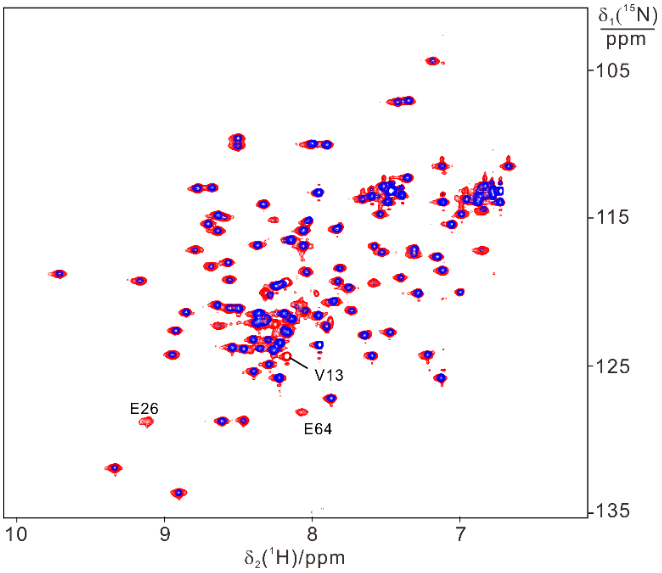

Figure S9. Interaction of BIR1 with copper sulfate is not buffer dependent. Superimposition of ^{15}N-HSQC spectra recorded for 0.1 mM D71N/R72E mutant (red) and after addition of 0.06 M $CuSO_4$ (blue) in 20 mM phosphate buffer at pH 7.0. NMR spectra were recorded in 20 mM phosphate buffer at pH 7.0 and 298 K with a proton frequency of 600 MHz.

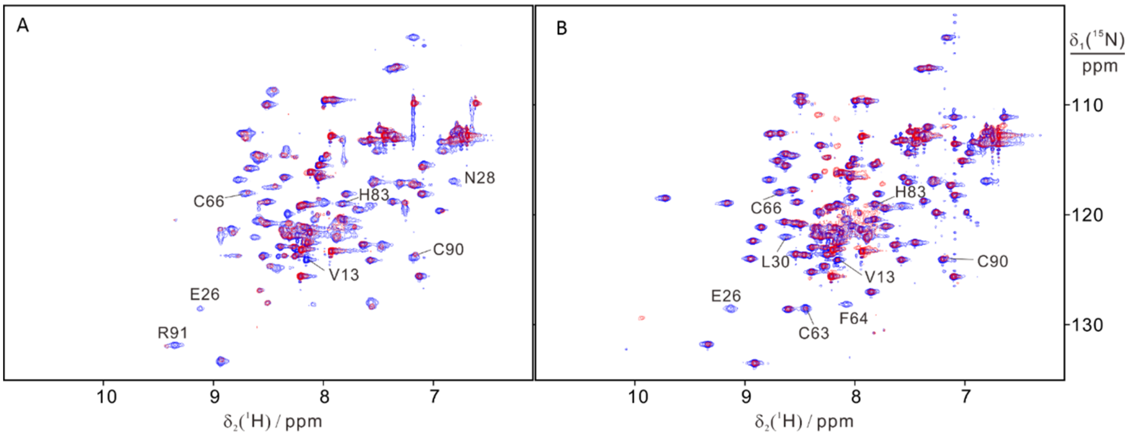

Figure S10. Superimposition of ^{15}N-HSQC spectra recorded for 0.15 mM BIR1 in the absence (blue) and presence of one equivalent of Cu(I) (red). (A) WT BIR1, (B) D71N/R72E mutant. NMR spectra were recorded in 20 mM Bis-Tris at pH 6.5 and 298 K with a proton frequency of 600 MHz and protein samples were made in glove box without O_2, and Cu(I) stock solution was used as 50 mM $[Cu(I)(CH_3CN)_4][PF_6]$ in CH_3CN.

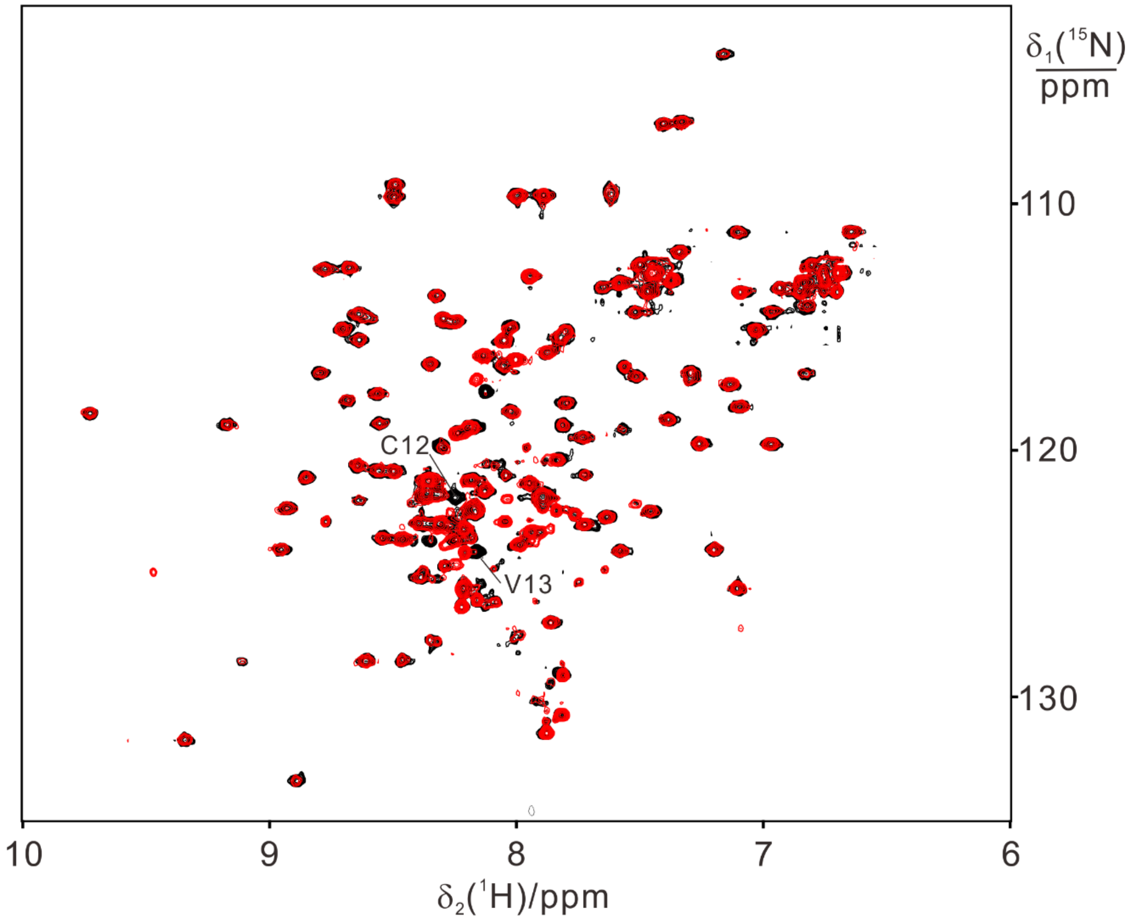

Figure S11. Interaction of D71N/R72E mutant with copper sulfate in *E. coli* cell lysate. Superimposition of ^{15}N-HSQC spectra recorded for cell lysate of D71N/R72E mutant (black) and after treatment with 1.0 mM $CuSO_4$ (red).

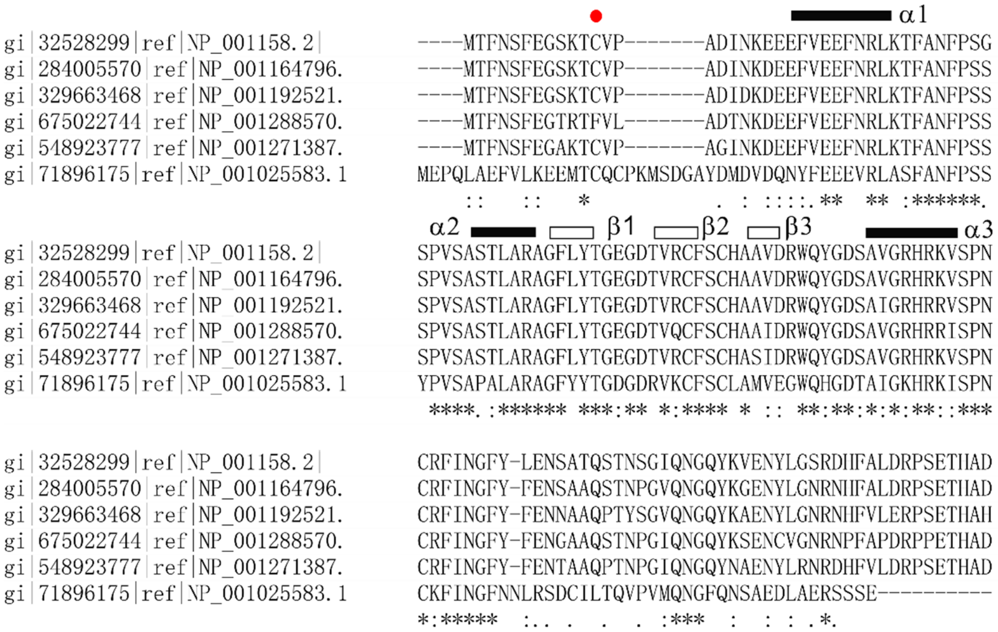

Figure S12. Structure based sequence alignment of XIAP BIR1 domain. The amino acid sequence of BIR1 (from the top to bottom) was from human, Rat, cow, mouse, dog and *Xenopus laevis*, respectively.

Table S1. Determined redox potential of Cys12 in wild type mutant by measuring the cross-peak intensity of Val13 in the reduced and oxidized BIR1 with respective concentration of DTT in 100 mM PBS at pH 7.0 and 298K.

$[DTT_{red}]_o$	$[WT_{ox}]$	$[DTT_{red}]$	$[WT_{red}]$	$[DTT_{ox}]$	E^0(V)
mM	mM	mM	mM	mM	
0.12	0.11	0.04	0.16	0.08	-0.3126
0.1	0.12	0.02	0.16	0.08	-0.3015
0.08	0.16	0.03	0.10	0.05	-0.2990

Table S2. Determined redox potential of Cys12 in BIR1 D71N/R72E by measuring the cross-peak intensity of Val13 in the reduced and oxidized BIR1 with respective concentration of DTT in 100 mM PBS at pH 7.9 and 298K.

$[DTT_{red}]_o$	$[DR_{ox}]$	$[DTT_{red}]$	$[DR_{red}]$	$[DTT_{ox}]$	E^0 (V)
mM	mM	mM	mM	mM	
0.12	0.13	0.03	0.19	0.09	-0.3045
0.1	0.15	0.02	0.17	0.08	-0.2941
0.08	0.18	0.02	0.13	0.06	-0.2890
